# Bioactive Materials and Novel Techniques in Endodontics: Translational Advances for Clinical Practice

**DOI:** 10.3390/jfb17020079

**Published:** 2026-02-06

**Authors:** Saulius Drukteinis, Matthias Widbiller, Sivaprakash Rajasekharan

**Affiliations:** 1 Institute of Dentistry, Faculty of Medicine, Vilnius University, Zalgirio 115, LT-08217 Vilnius, Lithuania; 2 Department of Conservative Dentistry and Periodontology, University Hospital Regensburg, Franz-Josef-Strauß-Allee 11, D-93093 Regensburg, Germany; matthias.widbiller@ukr.de; 3 Department of Paediatric Dentistry, School of Oral Health Sciences, Ghent University, B-9000 Ghent, Belgium; sivaprakash.rajasekharan@ugent.be

## 1. Context and Perspective

Over the past decade, endodontic biomaterials have shifted from being passive fillers to bioactive systems that can support repair and regeneration through validated physicochemical and biological mechanisms [1]. Advances in materials chemistry, nanotechnology, and bioengineering have now enabled contemporary materials to broaden therapeutic options and improve clinical performance [1,2,3]. Modern hydraulic calcium silicate-based (HCSB) sealers, bioactive resin-based materials, and nanostructured medicaments are designed to interact with host tissues via controlled ion release, mineralization, and cell-mediated responses, supported by extensive in vitro and in vivo evidence [1,2]. Simultaneous developments in regenerative endodontic strategies, including pulp revascularization and apexification, further demonstrate the clinical potential of biomaterials that combine multiple bioactive properties, such as odontogenic/osteogenic stimulation and sustained antimicrobial activity [4].

Despite significant progress, translating functional endodontic biomaterials from laboratory research to clinical practice remains challenging due to the complex root canal environment, including irregular anatomy, moisture, residual irrigants, microbial biofilms, and thermal or chemical stresses during obturation [5]. Long-term success depends on durable interactions at the biomaterial–tissue interface, including stable bonding to dentin, sustained antibacterial action, favorable physicochemical properties, and the desired biological effects of incorporated nanoparticles [3,6]. Therefore, to achieve predictable outcomes, integrated mechanistic in vitro studies complemented by long-term in vivo and clinical investigations are required [1,6].

HCSB materials continue to play a central role in contemporary endodontics due to their favorable biocompatibility, osteogenic potential, and sustained ion-release capacity [1,7]. Advances in fourth- and fifth-generation HCSB sealers and repair materials have improved their setting behavior, dimensional stability, and handling characteristics, facilitating broader clinical adoption [2,8]. Their defining advantage is intrinsic bioactivity—hydroxyapatite nucleation at the dentin–sealer interface—supporting long-term sealing and the “biological seal” concept [9,10]. This paradigm has also influenced obturation strategies, moving away from purely mechanical compaction toward biologically driven sealing concepts that exploit biomineralization and micromechanical interactions with dentin [8].

Despite these developments, antimicrobial efficacy remains a practical limitation for many commercially available HCSB sealers under clinically relevant conditions [11]. To address this, recent work has focused on modifying calcium silicate matrices with functional additives, including hydroxyapatite, cationic polymers such as PMETAC and chitosan, and ultra-small silver nanoparticles [12]. These modifications represent a transition toward nano-engineered HCSB systems designed to enhance antibacterial performance through controlled ion release and nanostructure-mediated interactions while maintaining compatibility with periapical tissues [3,11,12]. At the clinical level, the outcome data for a ready-to-use injectable calcium silicate-based sealer provide an important translational benchmark [13].

The emergence of functional HCSB sealers incorporating antibacterial nanoparticles and chitosan aligns obturation strategies with minimally invasive clinical approaches, including single-cone techniques, moisture-tolerant application, and simplified workflows [11,13]. However, successful clinical translation depends on standardized protocols that account for obturation temperature, irrigation regimens, and canal moisture, as these variables directly influence physicochemical and biological performance [13].

Effective root canal irrigation and intracanal medication are still fundamental to endodontic success, and current consensus emphasizes the importance of standardized protocols and clinically relevant endpoints [5]. Research is shifting from conventional disinfectants toward multifunctional agents with tissue-friendly profiles and bioactive properties, including combined antimicrobial and remineralizing potential [14]. Innovation in this field includes nano-enabled irrigants, optimized combinations of chelators/surfactants/nanoparticles, and activation systems to enhance debris removal, biofilm disruption, and dentin substrate integrity [5,14]. Although adjunctive activation methods have not always translated into definitive gains in long-term outcome, the emergence of novel irrigants and delivery concepts warrants continued clinically oriented research [5]. Intracanal medications are also shifting from calcium hydroxide pastes toward ready-to-use HCSB formulations that may be easier to remove, possess more antimicrobial properties in relevant settings, and be compatible with regenerative procedures [5]. Beyond temporary disinfection, medications may support apical repair and regeneration; for example, calcium silicate-based medications can modulate the release of dentin-derived growth factors such as TGF-β1 [15].

Regenerative endodontic procedures represent a fundamental shift from canal disinfection and sealing toward the restoration of the pulp–dentin complex. The central aspect of this approach is the tissue-engineering triad of stem cells, signaling molecules, and scaffolds, with scaffolds providing a permissive microenvironment for cell adhesion, differentiation, vascularization, and tissue integration [4]. Clinically used scaffolds include blood clots, platelet-rich plasma, and platelet-rich fibrin, and recent meta-analyses indicate that blood-clot scaffolds remain effective for increasing root length in immature teeth [16]. Increasing attention is being paid to engineered scaffolds (injectable hydrogels, fibrous matrices, decellularized extracellular matrix, and 3D-printed frameworks) that are better at controlling biological responses while maintaining mechanical stability [4].

Simultaneously, vital pulp therapy has become the increasingly accepted treatment compared with pulpectomy as indications and treatment options continue to expand. Clinical trials and systematic evidence consistently support bioactive HCSB cements over calcium hydroxide regarding the promotion of odontoblastic differentiation and dentin bridge formation [4,17]. Current innovations are also focused on resin–HCS hybrid materials that combine bioactivity with improved handling, mechanical strength, and esthetic stability [17]. However, the advantages of resin-based systems can come at the expense of solubility or ion exchange, which may attenuate aspects of bioactivity. The incorporation of nanofillers is also being explored to enhance antibacterial function and ion release while supporting pulp preservation across diverse clinical scenarios [3,17].

In pediatric endodontics, the materials must be biocompatible, (bio)resorbable, easy to handle, and quick to place, due to the developing tissues and limited cooperation [18,19]. The shift from formocresol pulpotomy to bioactive pulp capping and vital pulp therapy reflects a focus on preserving vitality and stimulating dentinogenesis [19]. Modern options—including resorbable calcium silicate cements, premixed injectable pastes, light-curable bioactive resins, and nanoparticle-enriched agents—aim to provide safer and more predictable outcomes while also reducing the time spent in the chair and improving acceptance among children [18,19].

Endodontic surgery remains a valid treatment option when nonsurgical retreatment is unsuccessful or contraindicated. Although the field of implant dentistry has expanded, well-designed comparative evidence indicates that surgical and nonsurgical endodontic retreatment can achieve survival rates comparable to, and in some instances exceeding, those of tooth replacements with dental implants [20]. The introduction of fourth- and fifth-generation HCSB root repair materials has further strengthened clinical predictability, with favorable physicochemical and biological properties and postoperative healing outcomes that are comparable to those achieved using mineral trioxide aggregate [1]. Commercial premixed, plasticized HCSB putties exhibit similar compositions and clinical performance, supporting their interchangeable use for root repair and root-end filling; nevertheless, procedure-dependent factors can alter pH and dislodgement resistance, underscoring the need for protocol-aware material evaluation [8].

The future of endodontic biomaterials is moving toward personalized, biologically guided therapy enabled by AI and predictive modeling [21]. As innovation accelerates—particularly for nano-enabled and regenerative systems—clinical translation will require rigorous standardization, regulatory harmonization, and clinically relevant testing, complemented by real-world evidence, post-market surveillance, and multicenter registries [21,22].

## 2. Special Issue Contributions

This Special Issue, “Advanced Materials for Clinical Endodontic Applications 2,” covers the transition toward bioactive endodontic biomaterials evaluated through clinically relevant and biologically meaningful endpoints. We have summarized the papers in this Issue below and related their findings to key developments shaping the broader field of endodontics. Together, they naturally cluster into three themes:

Sealer performance and safety: Huth et al. undertook an in vitro microscopical and microbiological assessment of the sealing ability of calcium silicate-based root canal sealers, providing clinically relevant evidence on sealing and microbial endpoints [23]. Aka et al. evaluated the cytocompatibility of endodontic bioceramics in human periodontal ligament-derived cells, strengthening the evidence concerning biological safety and host response [24]. Takahara et al. examined the biocompatibility and chemical properties of two bioceramic sealers in a rat molar sealer extrusion model, offering translational insights into tissue response under an extrusion scenario [25].

Bioactivity beyond obturation and regeneration-oriented platforms: Bilvinaite et al. demonstrated that a calcium silicate-based intracanal medicament can modulate TGF-β1 release from root canal dentine more effectively than calcium hydroxide, supporting the mechanistic rationale for biologically informed medicament selection [15]. Alghofaily et al. developed antibiotic-loaded chitosan–gelatin scaffolds for regenerative endodontic procedures and demonstrated a scaffold strategy that integrates biocompatibility with antibacterial activity [26].

Protocol sensitivity in root-end materials: Khedmat et al. investigated retro-cavity preconditioning (with and without EDTA) and its effects on root surface pH and the dislodgement resistance of calcium silicate-based retro-fills, highlighting how procedural steps can alter material performance and should therefore be explicitly reported and standardized [27].

Collectively, these contributions strengthen the case for protocol-aware material evaluation and for clinically relevant, biologically meaningful endpoints as the field advances from conventional obturation concepts toward responsive, clinically adaptable biomaterials.

## Data Availability

Not applicable.
